# Rheological and Chemical Evolution of HMA and WMA Binders Based on Ageing Kinetics

**DOI:** 10.3390/ma15020679

**Published:** 2022-01-17

**Authors:** Ao Huang, Gang Liu, Virginie Mouillet, Saannibe Ciryle Somé, Tingwei Cao, Haoliang Huang

**Affiliations:** 1 State Key Laboratory of Silicate Materials for Architectures, School of Materials Science and Engineering, Wuhan University of Technology, Wuhan 430070, China; ha@whut.edu.cn (A.H.); ctw-123@163.com (T.C.); 2 Cerema, Research Team DIMA, CEDEX 3, 13593 Aix-en-Provence, France; 3 Cerema, Research Team DIMA, 77171 Sourdun, France; ciryle.some@cerema.fr; 4 School of Materials Science and Engineering, South China University of Technology, Guangzhou 510000, China; huanghaoliang@scut.edu.cn

**Keywords:** bitumen, warm agent, foaming, ageing, rheology

## Abstract

It is fundamental to predict or estimate the rheological behavioural evolutions of binders and mixture to ensure a durability service life of the whole infrastructure. This study compared the long-term ageing kinetics of hot mix asphalt (HMA) and warm mix asphalt (WMA) produced with the same base bitumen. The difference in the component was that the WMA contained 1% of Cecabase warm agent and 5.5% of water by the weight of bitumen, to obtain a large expansion ratio (47 times). Rolling thin-film oven test (RTFOT) and pressure ageing vessel (PAV) laboratory ageing were carried out on the binder with or without the warm agent. The oven ageing procedure was conducted on the loose HMA and WMA mixtures for 0, 3, 6, and 9 days. Research results indicated that the dual effect of the studied warm agent and the foaming water sharply decreased the viscosity of the binder at a high temperature. Compared with the HMA, the warm agent improved the ageing resistance of the asphalt binder. However, higher content, such as 5.5 wt.%, of foaming water deteriorated viscosity due to a thinner bitumen film, which was more susceptible to oxidation. Therefore, less than 2 wt.% of warm agent and foaming water was recommended in the foamed WMA preparation.

## 1. Introduction

Warm mix asphalt has a paving temperature of 100–140 °C, lower by 20–40 °C than that of traditional hot mix asphalt (HMA) [1]. Compared with HMA, WMA can reduce CO_2_ emissions by an average of 67% [2] and save energy by about 30% [3]. There are three main methods to produce WMA by using (i) organic additives, (ii) chemical additives, and (iii) foaming techniques [4]. Wax or fatty amides are usually used as organic additives to reduce the viscosity of asphalt binder [5]. Chemical additives are usually a combination of emulsifiers or surfactants to help asphalt binder covers aggregate at a lower temperature [6]. In the foaming technique, the addition of water into the hot bitumen causes steam to entrap and generates a large volume of foam, which temporarily increases the volume of the asphalt binder and reduces mix viscosity [4].

The effect of each warm agent on asphalt binder property is different. Aspha-min and Sasobit agents improved the rutting resistance and crack resistance of binders but had no significant effect on fatigue performance [7]. Xiao indicated that the four types of WMA agents (Cecabase, Evotherm, Rediset, and Sasobit) after the RTFOT ageing still maintained viscoelastic properties of the binder [8]. Compared with the organic additives, the chemical additives were less affected by oxidative ageing [9]. The chemical additive Cecabase increased the stiffness of the binder by 1.3 times and imparted the WMA with more than 80% ability of HMA’s to resist the moisture damage [10]. Non-foaming additives demonstrated better ageing indices than foaming additives, and the chemical WMA additive exhibited better performance, compared with organic additives [11].

The water-based foaming techniques had excellent cost effectiveness and were the most widely used [12]. The binder foamed by 2% water exhibited a better ageing resistance for the short term but not for the long term [13]. The laboratory-prepared foamed WMA mixtures with 1.8% water underwent comparable or slightly lower levels of ageing, compared with traditional HMA mixtures [14]. It was indicated that the water content influenced the ageing properties of foamed WMA. The combination of chemical and foaming technology was also used to lower the construction temperature of the HMA. Firstly, a chemical additive was added into the hot coarse aggregates, and then, the wet sand was added to create a foaming action [15].

HMA and WMA consist of aggregates, bitumen, filler, and additives [16]. Their global mechanical performances entirely depend on their component’s percentage, the properties with the time evolution, and their interaction with each other [17]. During the design phase of the mixtures, it is fundamental to predict or at least estimate the evolution of rheological behaviours of binders and mixes to ensure the durability of the whole infrastructure [18]. In particular, WMA has more complex components, its mixing and paving temperatures are different from those of HMA, and its ageing protocols are still under investigation [19].

This study addresses the long-term ageing kinetic comparison of HMA and WMA manufactured with the same base bitumen. The results can be used to discriminate the ageing behaviour of WMA, compared with HMA. The paper focuses on analysing the evolution of physicochemical and rheological parameters of binders and mixes according to the long-term ageing kinetics. It also allows a reasonable ageing protocol to be proposed for foamed WMA, by comparing the RILEM ageing protocol on loose mixes following CEN TS 12697-52 and the rolling thin-film oven test (RTFOT), plus the pressure ageing vessel (PAV) method on bitumen following the EN 12607-1 and the EN 14769 developed for HMA.

## 2. Materials and Methods

### 2.1. Materials

The virgin binder was a paving grade bitumen 50/70 defined according to the European standard (EN 12591). It was provided by Nynas company (Stockholm, Sweden) and called Nyfoam since it was suitable to produce foam mix asphalt. It had a needle penetration value of 57 × 0.1 mm and a softening temperature of 49.6 °C according to EN 1426 and EN 1427.

Porphyry aggregates from Pont de Colonne Quarry (Côte-d'Or, France), were used. It had a Los Angeles abrasion loss (EN 1097-2) and microDeval fragmentation (EN 1097-1) of 17% and 8%, respectively.

Following the EN 13108-1, the hot mixture AC10 (HMA) was prepared with an optimum binder content of 5.4% at 165 °C. This mixture was mainly used for surface and binder courses of the pavement [20]. The Wirtgen WBL 10S (Wirtgen, Windhagen, Germany) equipment was employed to produce the foamed WMA at 135 °C. Compared with the HMA, the difference in the component was that the WMA contained 1% of Cecabase warm agent and 5.5% of water by the weight of bitumen, to obtain the maximum expansion ratio of the foam equal to 47 times of the initial bitumen volume.

### 2.2. The Ageing Process

RTFOT and PAV were used to simulate the short-term and the long-term ageing on asphalt binder, respectively. The related norms were EN 12607-1 and EN 14769. The WMA binder was prepared by mixing the base binder with 1 wt.% of the warm agent at 160 °C for 15 min. The RTFOT was performed on the WMA binder at 140 °C. Other parameters remained the same for the HMA binder according to the norms.

**Figure 1:**
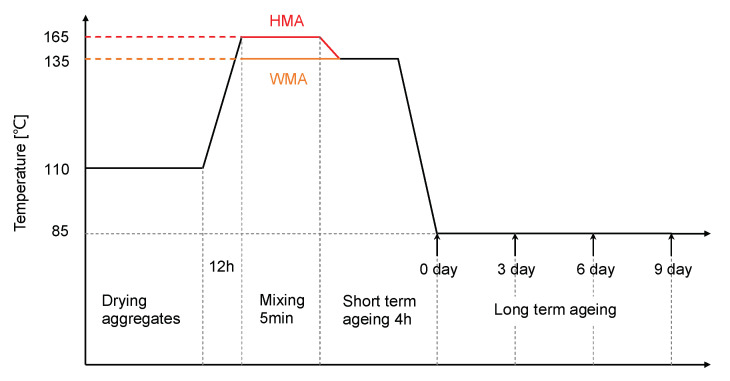
The illustration on the ageing protocols for the HMA and WMA mixtures.

The ageing protocol was performed on a loose asphalt mixture since it provided more explosion to the air which accelerated ageing, and meanwhile, the resulting ageing on the mixture was more homogeneous. After the mixing, the loose HMA mixture was cooled down from 165 °C to 135 °C and then spread in a tray with a thickness of around 2.5 cm. Then, the tray was placed in a ventilated oven for 4 h, to simulate the short-term ageing. Next, it was cooled down to 85 °C and kept in the oven for 3, 6, and 9 days, to simulate the long-term ageing. The WMA mixture was manufactured at 135 °C by the Wirtgen WBL 10S equipment and then immediately spread in the tray following the same short-term and long-term ageing procedure similar to the procedure performed on the HMA mixture. The ageing protocols on the HMA and WMA mixtures are illustrated in Figure 1, and the codes for the aged binder samples are given in Table 1.

### 2.3. Bituminous Binders Recovery

Binder extraction was performed according to the European standard NF EN 12697-3, and it was based on the dissolution in the dichloromethane solvent at room temperature. The obtained binder solution was then filtered and centrifuged to remove all aggregate particles from the binder solution. Afterwards, the solvent was removed by distillation under an air vacuum. It is important to note that after the binder was extracted and recovered, it was analysed by infrared spectroscopy to ensure complete solvent removal (no infrared peaks of chlorine), and the calcination residue during 8 h at 450 °C has to be less than 1% of binder test portion. After complete solvent removal, physicochemical and rheological studies were performed on recovered binders, aged at different levels from 0 to 9 days.

### 2.4. Testing Methods

**Figure 2:**
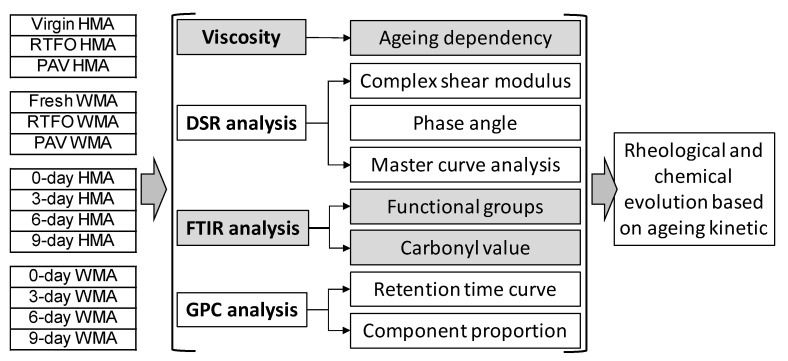
Detailed research program for rheological and chemical evolution study.

Figure 2 presents the detailed investigation program employed in this study to characterise the rheological and chemical evolution of both WMA and HMA binder based on ageing kinetics. Viscosity, DSR, FTIR, and GPC analyses were conducted for fundamental study on the slow ageing evolution of asphalt binder in the warm mix and hot mix, by means of both chemical study and mechanic test.

The dynamic shear rheometer (DSR), with the help of Anton Paar Smart Pave 102 (Graz, Austria), was adopted to characterise the complex modulus and phase angle of the asphalt binder. The test was performed at 20, 25, 30, 40, 50, and 60 °C, using the parallel plates with a diameter of 25 mm with a gap of 2 mm. Before starting the DSR measurement, strain sweeps were performed to determine the linear viscoelastic region of the binder. The WLF equation (WLF = William, Landel, and Ferry) was used to calculate the shift factors to construct master curves.

A cone/plate rheometer, with a cone angle of 1° and plate diameter of 25 mm installed on the DSR, was used to characterise the viscosity as a function of the shear rate. A shear rate sweep from 0.001 s^−1^ to 10 s^−1^ at 60 °C and 0.1 s^−1^ to 1000 s^−1^ at 120 °C was performed. 

A Thermo Scientific Nicolet 6700 Fourier Transform Infrared (FTIR) spectrometer (Thermo Fisher Scientific, Waltham, MA, USA) was used to characterise the chemical information of the binder at a middle infrared region of 2000~500 cm^−1^. Firstly, 0.1 g bitumen was dissolved in 2 mL carbon disulphide; next, the solution was applied on the KBr sheet; Finally, a Na lamp was used to dry the sheet. A carbonyl index (CI) was used to characterise the ageing degree quantitatively, and it was defined as

CI = A_1720–1678_/A_1400–700_

where A_1720–1678_ is the carbonyl area in the range of 1720–1678 cm^−1^, and A_1400–700_ the fingerprint area in the field of 1400–700 cm^−1^.

Gel permeation chromatography (GPC) was used to analyse the molecular weight distribution of virgin and aged binders [21]. The Agilent PL-GPC50 equipment (Santa Clara, CA, USA) was used with a chromatographic column of Agilent MIXED-C columns and a constant temperature of 40 °C. Testing parameters are given in Table 2.

**Figure 3:**
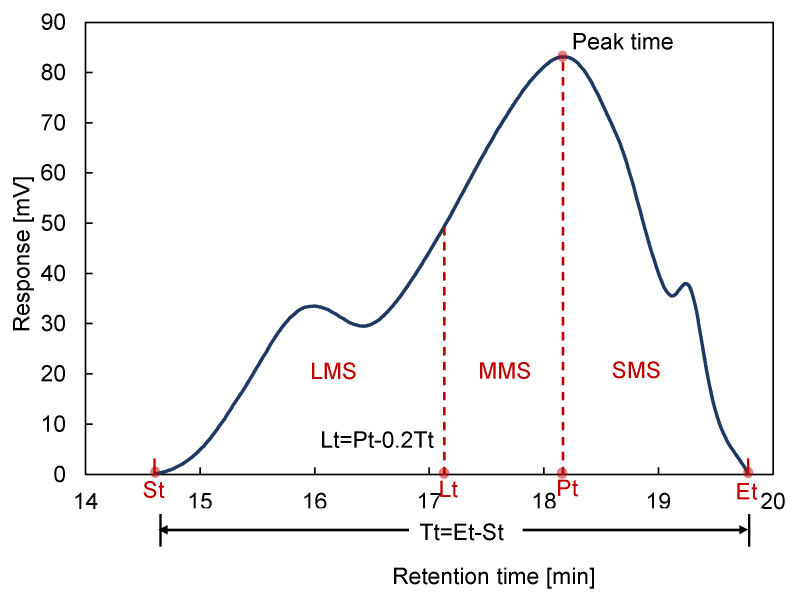
Illustration of determining LMS time (Lt) from total retention time (Tt) of the chromatogram of virgin binder (asphalt A), based on ending time (Et) and starting time (St) [22].

Based on the GPC results, bituminous molecules were divided into three types with a small molecular size (SMS), a medium molecular size (MMS), and a large molecular size (LMS), which are illustrated in Figure 3. The area of LMS, MMS, and SMS underneath the GPC curve represented the related quantity and was calculated for further study.

The IATROSCAN MK-6S rod thin layer chromatography (NTS America, Inc., Brooklyn, NY, USA) was adopted to determine four components of binder in this study [23]. First, the bitumen/dichloromethane solution was configured with a mass concentration of 20 mg/mL. Next, a micro-syringe was used to transfer 50 µL of the solution to the origin of the silica gel chromatographic rod and place it in the cylinder at a constant temperature to dry. Finally, four components were separated as follows:

(1) The n-heptane was used, to expand the four components in the rod to the chromatographic bar scale of 100, and dried at 80 °C for 1 min;
(2) A mixed solvent of toluene and n-heptane (volume ratio 1:1) was used, to expand the components to the chromatographic bar scale of 50, and dried at 80 °C for another 1 min;
(3) The mixed solvent of toluene and ethanol (volume ratio 1:1) was used, to expand the components to the chromatographic bar scale of 25, and dried at 80 °C for 1 min;
(4) Finally, the rod was placed in hydrogen flame condition at a constant speed to measure the weight of the four individual components.

## 3. Results

### 3.1. Viscosity

**Figure 4:**
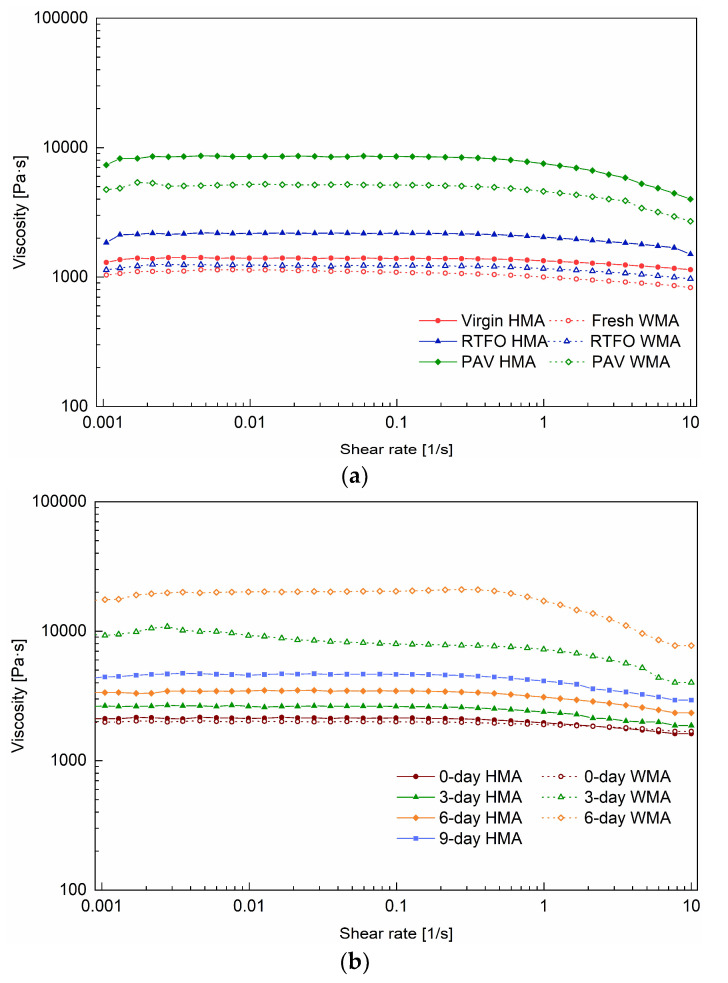
Shear rate dependency of viscosity of the binders at 60 °C before and after ageing: (**a**) for groups 1, 2, and (**b**) for groups 3 and 4.

With the shear rate ranging from 0.001 s^−1^ to 1 s^−1^, a Newtonian plateau was observed for all binders, and their zero-shear viscosity was obtained. In comparison, the zero-shear viscosity of fresh WMA binder was lower than the virgin HMA binder, due to the addition of a warm agent. The zero-shear viscosities of 0-day HMA and 0-day WMA binders were very close and higher than that of virgin HMA. This means that ageing occurred while producing the HMA and WMA mixtures. When the shear rate was higher than 1 s^−1^, the shear-thinning behaviour was observed and was more evident for aged binders, such as PAV HMA, PAV WMA (Figure 4a), 3-day WMA, and 6-day WMA binders (Figure 4b). Shear thinning occurs because the macromolecule chains usually exist in a state of random orientation and high entanglement, and they will become disentangled and oriented at high shear rates. It was revealed that ageing increased the content of macromolecules in the bitumen.

**Figure 5:**
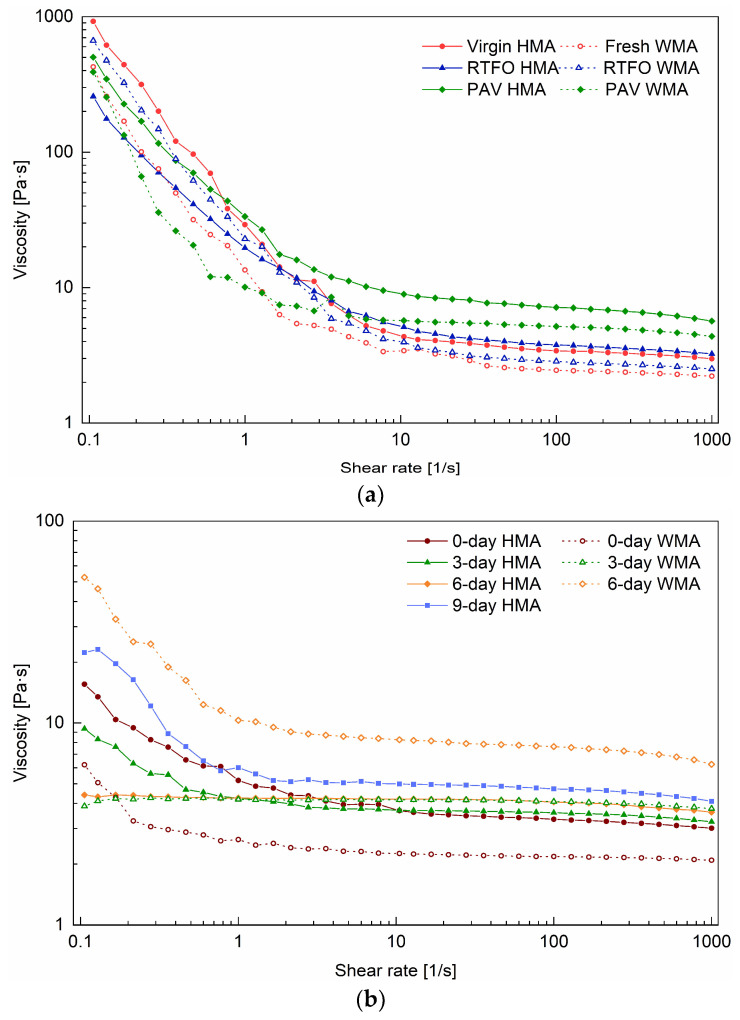
Shear rate dependency of viscosity of the binders at 120 °C before and after ageing: (**a**) for groups 1, 2, and (**b**) for groups 3 and 4.

Figure 5 shows the shear rate dependency of viscosity of the studied binders at 120 °C before and after ageing. Different from the curve at 60 °C, a shear thinning of the bitumen started at a low shear rate, and a Newtonian plateau was reached above 10 s^−1^. The shear thinning for binder groups 1 and 2 (Figure 5a) was more obvious than that for binder groups 3 and 4 (see Figure 5b) between the shear rates of 0.1 s^−1^ and 10 s^−1^. The reason could be that the extraction process with asphalt mixture made the bituminous molecules more oriented [24]. The ageing conditions on the binder and mixture could be another reason to cause the differences in this behaviour.

Table 3 lists the viscosity of binders at 60 °C and 120 °C. The viscosity at the corresponding shear rates belonged to the Newtonian plateau and can reflect the intrinsic behaviour of the asphalt binder. The viscosity of 2.18 Pa·s for 0-day WMA binder was less than 3.33 Pa·s for 0-day HMA binder. With the addition of a warm agent, the ageing resistance of the binder was improved. Compared with the virgin HMA binder, the PAV WMA binder had a viscosity of 5.17 Pa·s less than 7.15 Pa·s for the PAV HMA binder. However, the 6-day WMA had a viscosity of 7.63 Pa·s, much higher than 4.06 Pa·s for the 6-day HMA binder. The reason could be that the addition of water accelerated the ageing of the mixture whose film thickness was thinner than the mixture aged in RTFOT and PAV. The viscosity of the 9-day WMA was too high to be measured.

### 3.2. Viscoelastic Properties

**Figure 6:**
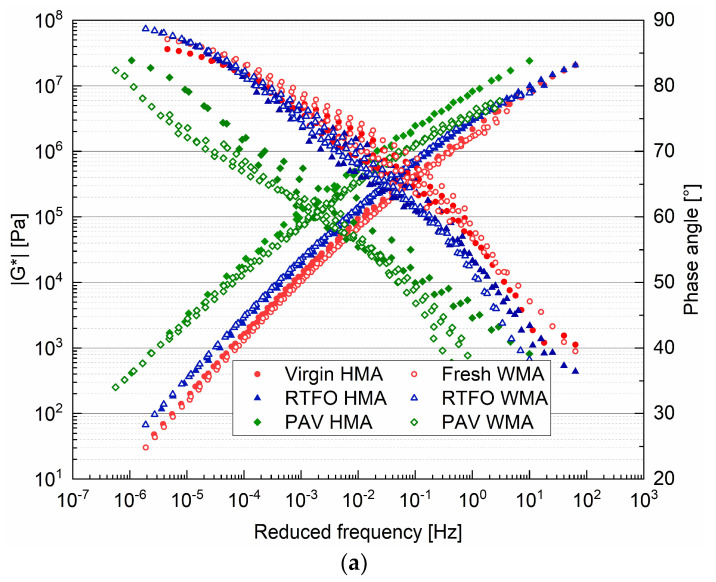

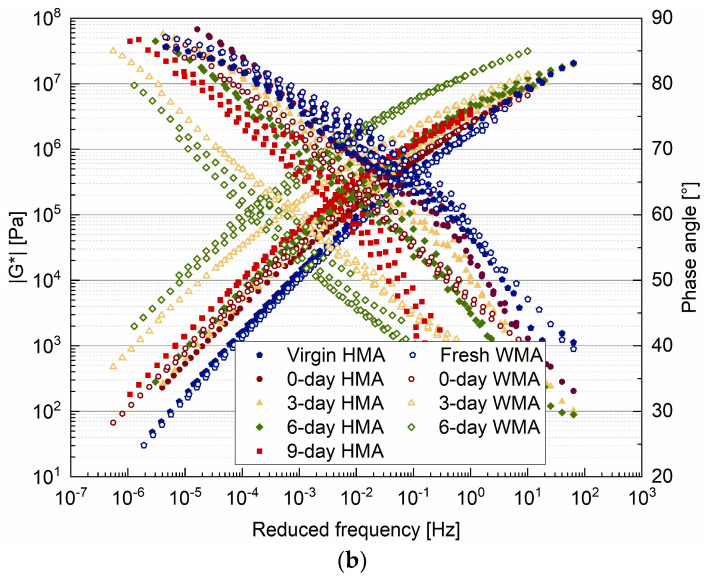
Master curve of (**a**) HMA binders and (**b**) WMA binders before and after ageing. Ascending curves represent complex moduli |G*| while descending curves represent the phase angle.

For the binders shown in Figure 6, a general observation was that the complex modulus of the binders converged at the high-frequency region, and the phase angle converged at the low-frequency area. Due to the addition of the warm agent, the fresh WMA binder had a slightly lower complex modulus and a higher phase angle than the Virgin HMA binder (Figure 6a). In comparison, the modulus master curve of the PAV WMA binder was located below that of the PAV HMA binder. This means that the warm agent used in this study improved the ageing resistance of the virgin binder.

As shown in Figure 6b, the modulus master curve of the 0-day HMA binder was located above that of the virgin HMA binder due to short-term ageing, as illustrated in Figure 1. The complex modulus of binders gradually increased with ageing time. The complex modulus of the 0-day WMA binder was very close to that of the 6-day HMA. It was found that the binder in the WMA mixture quickly aged in the oven. The 6-day WMA binder had the highest complex modulus and the lowest phase angle. The 9-day WMA binder was too hard to undergo experiment by the frequency sweep.

Typically, water content less than 2% by the weight of binder was used in the foam asphalt mixture. Previous research indicated that a water content higher than 2% accelerated the ageing of the binder [25]. The high water content of 5.5% caused a large expansion ratio (47 times). Then, it made the bitumen film thinner and more susceptible to oxidation. Another point worth noting is that the positive effect of warm mixes on the long-term performance of asphalt was no longer present.

### 3.3. FTIR

**Figure 7:**
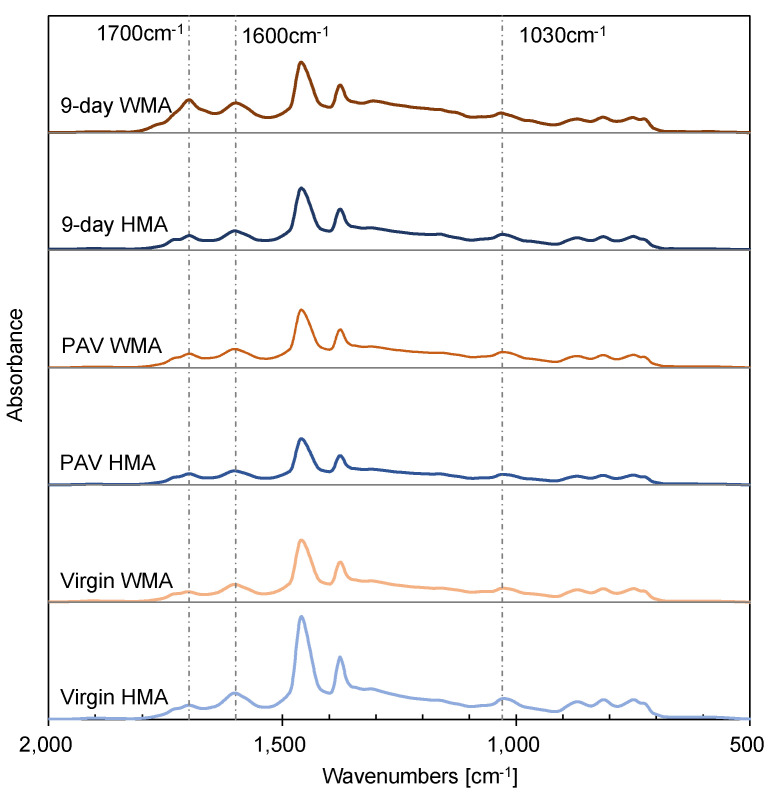
FTIR spectra for the WMA binders before and after ageing.

As shown in Figure 7, FTIR was used to evaluate the ageing behaviour of HMA and WMA binders. Typical peaks were observed, such as in-plane bending vibration of the –OH groups at 1627 cm^−1^ and the scissoring vibration of –CH_2_ at 1464 cm^−1^. There was no specific characteristic peak for the warm agent when comparing fresh WMA and virgin HMA binders. Two critical functional groups were characterised by the FTIR spectra. One was the carbonyl group at 1700 cm^−1^, and another was the sulphoxide group at 1030 cm^−1^. Both belong to the stretching vibration and are formed due to the oxidation, dehydrogenation, and crosslinking reactions occurring simultaneously. The carbonyl group was more sensitive to ageing in this study, and its band became broader as ageing proceeded. Therefore, the CI value was used to characterise the ageing degree semi-quantitatively.

**Figure 8:**
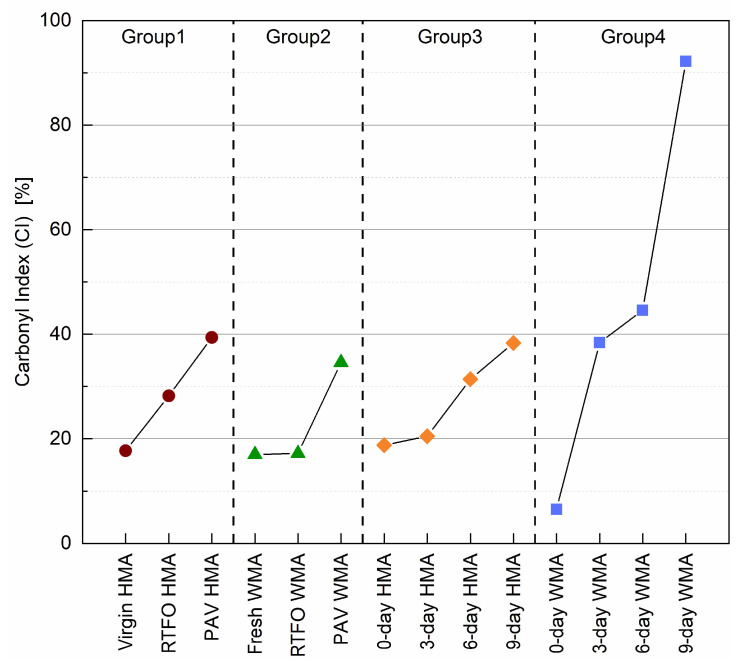
The carbonyl index (CI) for four binder groups before and after ageing.

Figure 8 shows a clear evolution of ageing for four binder groups based on their CI value. The more severe the oxidation reaction carried out, the more carbonyl groups were formed. Before ageing, the binders of virgin HMA, fresh WMA, 0-day HMA, and 0-day WMA had a comparable CI value. As ageing proceeded, the CI value for the virgin HMA binder in group 1 increased linearly, from 17.8% to 39.4%. However, the CI value for the fresh WMA binder in group 2 almost did not change after RTFO ageing.

In comparison, the CI value for the PAV WMA binder in group 2 was lower than that of the PAV HMA binder in group 1. The reason could be that the addition of the warm agent increased the ageing resistance of the virgin binder. Binder group 3 underwent mixture oven ageing, and its 9-day HMA binder had a comparable CI value with that of the PAV HMA binder. However, the CI value of binders in group 4 increased rapidly as ageing proceeded, which means that the oven ageing of the foamed binder in the mixture accelerated with a water content of 5.5%.

### 3.4. GPC

**Figure 9:**
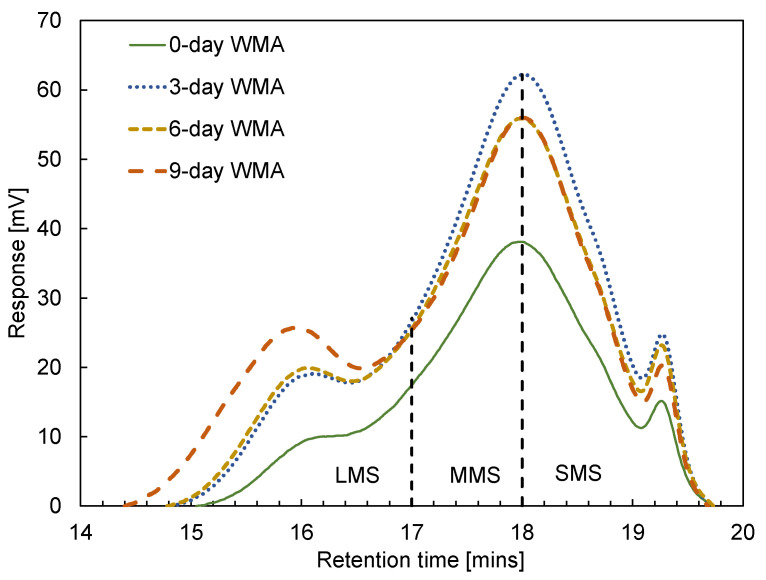
GPC curves for the binders of group 4.

Figure 9 shows a typical example of GPC curves for the binders in group 4. There are mainly three peaks from left to right for each curve, representing LMS, MMS, and SMS parts. For the binder from 0-day WMA to 9-day WMA, three main peaks shift to the left. The width of the first peak represents the part of LMS increased, which means that the bituminous molecules were transformed from small to large ones due to ageing.

**Figure 10:**
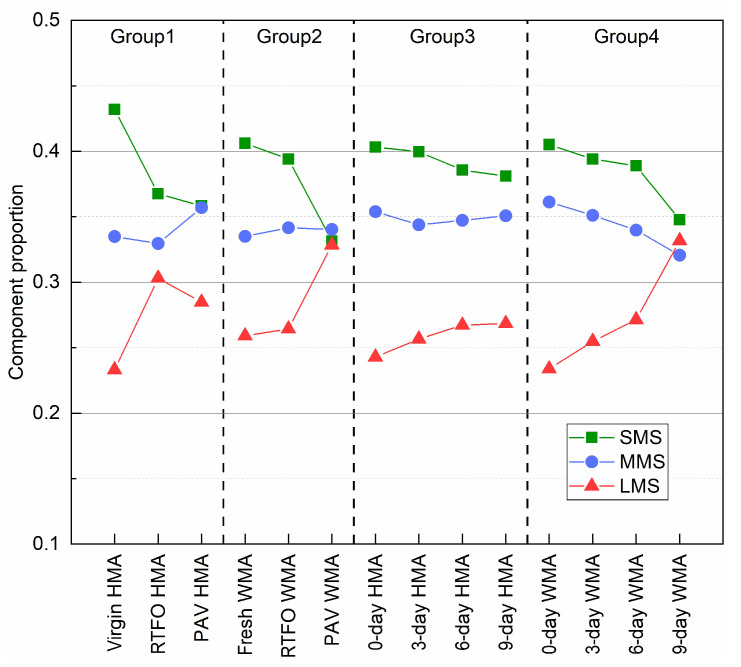
Components proportion (SMS, MMS, and LMS) of the binder before and after ageing according to the GPC curve.

Figure 10 shows the change in the component proportion of each binder due to ageing. In general, the SMS proportion decreased, but that of the LMS increased as ageing proceeded. However, the changing trend in the MMS proportion depended on the binder group and ageing condition. Volatilisation and oxidation simultaneously occurred when preparing fresh WMA, 0-day HMA, and 0-day WMA binders. Therefore, they had a lower SMS and a lower LMS than the virgin HMA binder. The LMS component increased sharply for binder group 2, and three components became comparable after PAV ageing. Three components for group 3 had a slight change as ageing proceeded. Even after the 9-day oven ageing, the LMS component only increased by 9.3%. This means that the HMA mixture had better ageing resistance. For group 4, both SMS and MMS decreased, and the LMS dramatically increased to 45.8%.

### 3.5. The Four Fractions

**Figure 11:**
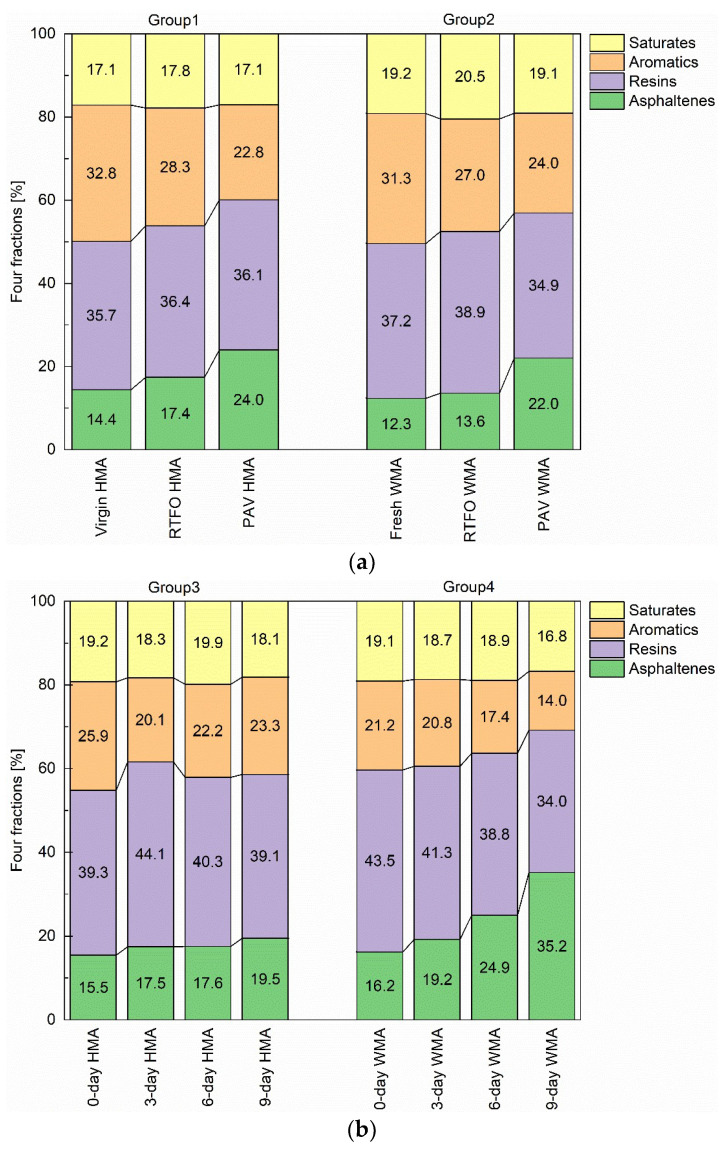
Change in four fractions of all binder as a function of ageing. (**a**) for groups 1, 2, and (**b**) for groups 3 and 4.

Figure 11a shows the change in four fractions of all binders as a function of ageing. After PAV ageing, the aromatics content for the virgin binder in group 1 decreased by 10%, but its asphaltenes content increased by almost the same percentage. However, its saturates and resins contents were relatively stable. It means that after PAV ageing, the transformation of aromatics to resins equalled resins to asphaltenes. After the same ageing mode (RTFOT or PAV), the contents of resins and asphaltenes in group 2 were lower than those in group 1. This means that the addition of the warm agent improved the ageing resistance of the virgin binder.

For group 3, its asphaltenes content increased slightly as oven ageing proceeded (Figure 11b). However, the changing trend for aromatics and resins fluctuated. For example, there was a decrease in resins but an increase in aromatics after the 6-day oven ageing. The reason could be that decomposition occurred on resins [26]. For group 4, there was a clear changing trend for each fraction. After 9-day oven ageing, aromatic and resins were continuously transformed towards asphaltenes, which reached 35.2%; the value of asphaltene confirmed its considerable ageing condition.

The colloid index (Ic) was employed to describe ageing behaviour. It is defined as the ratio of the dispersed component to the flocculating component as follows:

Ic = (Aromatics + Resins)/(Saturates + Asphaltenes)(1)

**Figure 12:**
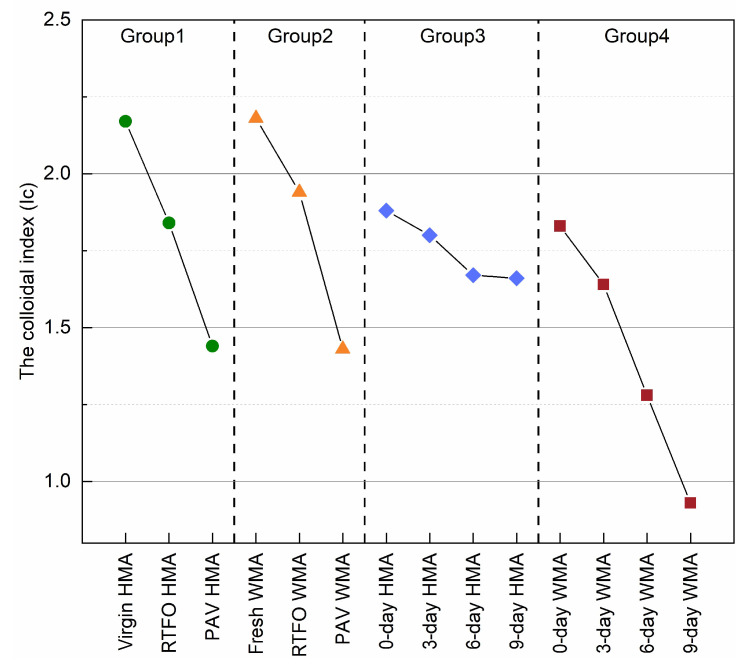
Change in Ic of all binder as the function of ageing.

A higher Ic value means a lower ageing degree of binder or better colloidal stability. As Figure 12 shows, the Ic value of fresh WMA and RTFO WMA was slightly higher than those of virgin HMA and RTFO HMA. This result means that the addition of a warm agent improved its short-time ageing resistance. The Ic value of 0-day HMA was lower than that of virgin HMA, which means that the foaming process destroyed the colloidal performance of the binder. However, its Ic value changed slightly from 0-day to 9-day oven ageing. The Ic value for group 4 decreased sharply and reached a minimum of 0.93 after 9-day oven ageing. This means that the WMA mixture had a worse ageing behaviour than the HMA mixture in this study.

A correlation (r) analysis was performed between the viscosity and chemical properties of all 14 binder samples. The results indicated that the viscosity at 60 °C had the highest correlation with the asphaltene content (r=0.81), followed by the colloid index (r = 0.78) and carbonyl content (r = 0.74) and the LMS area (r = 0.69).

## 4. Conclusions

Based on the rheological and chemical results, the following conclusions were drawn:

(1) The warm agent reduced the viscosity of bitumen and its shear-thinning behaviour. The dual effect of the used warm agent and foaming water reduced the viscosity of bitumen by 34.5% at 120 °C. It can, therefore, effectively reduce the rolling temperature of its corresponding asphalt mixture, improving its compaction efficiency and saving energy consumption.
(2) The viscosity of the binder, as an indicator of the evolution of ageing with time, had the highest correlation with the asphaltene content, followed by the colloid index, the carbonyl content, and the LMS area. Such value with high correlation can be used as one of the standards to quickly judge the degree of asphalt ageing.
(3) Compared with HMA, the warm agent improved the ageing resistance of the binder. However, the high foaming water content of 5.5% deteriorated this property during oven ageing on the mixture. The reason could be that a large expansion ratio makes the bitumen film thinner and more easily reachable by oxygen and then resulted in more oxidation. Based on the results and previous experience, less than 2% or warm agent and foaming water is recommended as the optimal ratio for foamed WMAs.

## Figures and Tables

**Table 1:** Codes for the binder samples before and after ageing.

Binder Group	Binder Code	Items
Group 1	Virgin HMA	Virgin Nyfoam binder
	RTFO HMA	after RTFOT at 163 °C
	PAV HMA	after RTFOT at 163 °C + 20 h PAV
Group 2	Fresh WMA	Virgin Nyfoam binder + 1% warm agent
	RTFO WMA	Fresh WMA binder after RTFOT at 140 °C
	PAV WMA	Fresh WMA binder after RTFOT at 140 °C + 20 h PAV
Group 3	0-day HMA	Recovered from the loose HMA mixture
	3-day HMA	Recovered from the HMA mixture after 3-day oven ageing
	6-day HMA	Recovered from the HMA mixture after 6-day oven ageing
	9-day HMA	Recovered from the HMA mixture after 9-day oven ageing
Group 4	0-day WMA	Recovered from the loose WMA mixture
	3-day WMA	Recovered from the WMA mixture after 3-day oven ageing
	6-day WMA	Recovered from the WMA mixture after 6-day oven ageing
	9-day WMA	Recovered from the WMA mixture after 9-day oven ageing

**Table 2:** GPC test conditions for bitumen.

Items	Content
Mobile phase	Tetrahydrofuran (THF)
Solvent (sample preparation)	THF (30 min, 100 rpm at room temperature)
Sample concentration	0.10 mg/mL
Injection volume	100 µL
Flow rate	1.5 mL/min
Test temperature	40 °C
Detector	Photodiode Array, (PDA)
Columns	Agilent PLgel 5 µm MIXED-C columns
Calibration standards	Polystyrene

**Table 3:** Viscosity of all binders at 60 °C and 120 °C.

Binder Groups		Viscosity at 60 °C and 1 s^−1^ (Pa·s)	Viscosity at 120 °C and 100 s^−1^ (Pa·s)
Group 1	Virgin HMA	1337.4	3.42
	RTFO HMA	2035.20	3.77
	PAV HMA	7524.70	7.15
Group 2	Fresh WMA	1000.80	2.45
	RTFO WMA	1163.60	2.85
	PAV WMA	4596.00	5.17
Group 3	0-day HMA	1994.20	3.33
	3-day HMA	2426.70	3.59
	6-day HMA	3171.10	4.06
	9-day HMA	4222.80	4.71
Group 4	0-day WMA	1912.80	2.18
	3-day WMA	7415.10	4.08
	6-day WMA	18445.00	7.63

## Data Availability

The reported results of supporting data can be found on the Web of Science.
